# Dexamethasone Promotes *Aspergillus fumigatus* Growth in Macrophages by Triggering M2 Repolarization via Targeting PKM2

**DOI:** 10.3390/jof7020070

**Published:** 2021-01-20

**Authors:** Maureen K. Luvanda, Wilfried Posch, Jonathan Vosper, Viktoria Zaderer, Asma Noureen, Cornelia Lass-Flörl, Doris Wilflingseder

**Affiliations:** 1 Institute of Hygiene and Medical Microbiology, Medical University of Innsbruck, 6020 Innsbruck, Austria; maureen.luvanda@i-med.ac.at (M.K.L.); wilfried.posch@i-med.ac.at (W.P.); viktoria.zaderer@i-med.ac.at (V.Z.); asma.noureen@i-med.ac.at (A.N.); cornelia.lass-floerl@i-med.ac.at (C.L.-F.); 2 Institute of Medical Biochemistry, Medical University of Innsbruck, 6020 Innsbruck, Austria; jonathan.vosper@i-med.ac.at

**Keywords:** glucocorticoids, dexamethasone, macrophages, glycolytic pathway, PKM2, *Aspergillus fumigatus*

## Abstract

Since long-term corticosteroid treatment is associated with emerging opportunistic fungal infections causing high morbidity and mortality in immune-suppressed individuals, here we characterized the impact of dexamethasone (Dex) treatment on *Aspergillus fumigatus*-related immune modulation. We found by high content screening and flow cytometric analyses that during monocyte-to-macrophage differentiation, as little as 0.1 µg/mL Dex resulted in a shift in macrophage polarization from M1 to M2-like macrophages. This macrophage repolarization mediated via Dex was characterized by significant upregulation of the M2 marker CD163 and downmodulation of M1 markers CD40 and CD86 as well as changes in phenotypic properties and adherence. These Dex-mediated phenotypic alterations were furthermore associated with a metabolic switch in macrophages orchestrated via PKM2. Such treated macrophages lost their ability to prevent *Aspergillus fumigatus* germination, which was correlated with accelerated fungal growth, destruction of macrophages, and induction of an anti-inflammatory cytokine profile. Taken together, repolarization of macrophages following corticosteroid treatment and concomitant switch to an anti-inflammatory phenotype might play a prominent role in triggering invasive aspergillosis (IA) due to suppression of innate immunological responses necessary to combat extensive fungal outgrowth.

## 1. Introduction

Macrophages are the predominant phagocytic cells in the pulmonary system arising from bone-marrow-derived monocyte precursors [1,2]. They are recruited to sites of infection, making them crucial first-line defenders within the innate immune system against bacterial and fungal pathogens that are able to breach the epithelial barrier [3,4,5]. Macrophages exhibit flexibility in their functional and phenotypic capacities, which are dictated by specific environmental stimuli [6]. They therefore exist as a heterogeneous population with a high degree of metabolic diversity [7,8]. Although macrophage phenotypes defy simple categorization and display a high degree of plasticity, they can be broadly grouped into two classes according to whether they are pro-inflammatory (M1) or anti-inflammatory (M2), with a corresponding negative influence in the context of fungal infections [9,10,11]. *Aspergillus (A.) fumigatus* is the most prevalent saprophytic fungal pathogen in immunocompromised individuals [12,13,14], and it invades both the upper and lower respiratory tracts, resulting in severe pulmonary diseases, such as IA [13,15,16,17].

Corticosteroids are crucial hormones endogenously produced by the adrenal glands to regulate essential inflammation-related processes [18,19], but their main function is to maintain homeostasis [20]. Corticosteroids can impinge on a variety of metabolic, reproductive, and developmental processes [21]. For that reason, they are applied in autoimmune disorders, allergic reactions, transplantation post-treatment therapy, and as a prophylaxis prior to chemotherapy [22,23]. Some treatment regimens demand high corticosteroid doses associated with various adverse off-target effects [23,24,25]. As a result, they are also a major predisposing factor for acquiring *A. fumigatus* infection [26,27,28]. The severity of these diseases is heavily dependent on the potency of the immunosuppressive regimen [29].

Among corticosteroids, Dexamethasone (Dex) and Prednisolone are given due to their excellent oral bioavailability, reduced mineralocorticoid activity, and easy elimination via renal excretion or hepatic metabolism [30]. However, their capacity to modify metabolic pathways in immune cells remains unknown, and thus, we here studied the impact of Dex on *Aspergillus*-related immunity and first-line defense mechanisms. Transcriptomic studies recently demonstrated that fungi such as *A. fumigatus* are capable of modulating the metabolic activities of macrophages by inducing increased glycolytic flux, therefore promoting inflammation [31]. The M2 isoform of Pyruvate Kinase (PKM2), an enzyme catalyzing the last step of glycolysis [25], has been reported to attenuate the M1 phenotype after activation in favor of a more M2-like macrophage concurrently inhibiting IL-1β production and boosting IL-10 secretion [32]. Many studies have implicated PKM2 and the glycolytic pathway as a potential therapeutic target for several inflammatory disorders, but the impact that corticosteroids have on this enzyme during opportunistic fungal infections remains unclear. Here, we found that even small concentrations of Dex during monocyte-to-macrophage differentiation shifted M1 into M2-like macrophages in a PKM2-dependent fashion and promoted fungal growth and concomitant macrophage infection as also observed during IA.

## 2. Materials and Methods

### 2.1. Ethics Statement

The use of anonymized specimens for scientific purposes was approved by the Ethics Committee of the Medical University of Innsbruck, and informed consent was obtained from all volunteer blood donors by the Central Institute for Blood Transfusion & Immunological Department, Innsbruck, Austria (ECS1166/2018, 14 November 2018).

### 2.2. THP-1 Cell Culture

To evaluate the range of physiologically relevant Dex and solvent (EtOH) concentrations not harming cell survival upon long-term culture, THP-1 cells (ATCC^®^; TIB—202^TM^, Manassas, VA, USA) were cultured in RPMI 1640 media supplemented with 10% fetal bovine serum (FBS), 1 × penicillin/streptomycin, 2 mM L-glutamine (all obtained from Sigma-Aldrich, St Louis, MO, USA) in a humidified incubator under 5% CO_2_ at 37 °C. THP-1 cells were stimulated into macrophages using 50 ng/mL phorbol 12- myristate 13-acetate (PMA), 1% sodium pyruvate, and 1% Eagle’s minimum essential medium with non-essential amino acids (all obtained from Sigma-Aldrich, St. Louis, MO, USA) and were simultaneously treated with different concentrations of Dexamethasone (purity > 99%, Sigma-Aldrich, St. Louis, MO, USA) and Ethanol (EtOH) (Carl Roth). More than 90% of the cells obtained expressed the CD68 lineage marker, indicating successful macrophage differentiation. Cells were cultured in a humidified incubator at 37 °C/5% CO_2_ for another 48 h.

### 2.3. Monocyte Isolation and Differentiation into MDMs

In view of the fact that cell lines have been reported to exhibit abnormalities at a chromosomal level [33], the main experiments were carried out using primary monocyte-derived macrophages. Peripheral Blood Mononuclear Cells (PBMCs) were separated from whole blood by density gradient centrifugation using Ficoll-PaqueTM Premium (GE Healthcare Europe, GmbH, DE) at 400× *g* for 15 min at room temperature, and then monocytes were isolated by magnetic bead separation using CD14 magnetic microbeads (BD Imag^TM^, San Diego, CA, USA) as previously described [34]. Identity and purity were confirmed by flow cytometry using a mouse-α-human CD14 antibody (Biolegend, San Diego, CA, USA), mouse anti-human CD3 FITC-labelled antibody (BD Pharmingen, Franklin Lakes, NJ, USA) and mouse anti-human CD19 APC-labeled antibody (Biolegend, San Diego, CA, USA). For differentiation into macrophages, monocytes were then cultured in RPMI 1640 media supplemented with 2.5% human AB serum and 2 mM L-glutamine (all obtained from Sigma-Aldrich, St. Louis, MO, USA) in a humidified incubator at 37 °C/5% CO_2_. They were then stimulated with 50 ng/mL rhGMCSF to obtain a M1-like phenotype and 50ng/mL rhMCSF to obtain a M2-polarized phenotype (both obtained from Biolegend, San Diego, CA, USA) with or without Dexamethasone. The cells remained in culture for 7 days (168 h) without any media replenishment during this period. In some experiments, differentiated macrophages were stimulated with *A. fumigatus* conidia.

### 2.4. Dexamethasone Dilution and Treatments

Human blood-derived monocytes were cultured in 6-well plates at a concentration of 1 × 10^6^/mL and treated with rhGM-CSF (50 ng/mL) plus Dex at 0.1 µg/mL, 0.5 µg/mL, 1 µg/mL, 100 µg/mL and 500 µg/mL. Two controls were included: 50 ng/mL rhGMCSF (GM-MDMs) and EtOH control stimulated with rhGMCSF and 2.4% Ethanol, corresponding to the highest EtOH concentration used (500 µg/mL Dex). Dexamethasone treatment was applied during the entire differentiation period of 7 days (T_168_).

### 2.5. Viability, Characterization, and Surface Marker Immunostaining for Expression Analysis

Macrophages were mechanically harvested after 7 to 12 days of differentiation/treatment using a cell scraper (Greiner Bio-one, Kremsmünster, AT), spun at 1000 rpm/10 min, and analyzed for cell integrity and viability by microscopy using Trypan blue exclusion or a Live/Dead cell viability dye (Ghost Dye^TM^ Violet 510, Tonbo Biosciences, San Diego, CA, USA). For immunophenotyping, viable cells were stained using specific fluorophore-conjugated anti-human monoclonal antibodies (Table 1) at concentrations recommended by the manufacturer. Cells were incubated with Abs for 30 min at +4 °C in the dark, washed using PBS/0.1% BSA (FACS Wash) buffer, and fixed in 4% Paraformaldehyde (Biolegend, San Diego, CA, USA) before analyzing at BD FACS Verse^TM^ flow-cytometer (BD Biosciences, San Diego, CA, USA). Live/dead dye was routinely applied to exclude dead cells before analyzing cells of interest. Data were analyzed using FACS Suite or Diva software v 9.0 (BD Biosciences, San Diego, CA, USA).

### 2.6. Aspergillus fumigatus Culture

The Ds Red-expressing isolate of *A. fumigatus* strain AF293 (Taxonomy ID: 330879) was the pathogen used for infection and stimulation [35]. It was cultivated in *Aspergillus* Complete Medium, as previously described [36], for 5 days at 35 °C in aerobic conditions. For harvesting, spore buffer solution (0.9% NaCl and 0.1% Tween 20) was poured onto the culture plates, and the fungi were scraped off with a sterile plastic wire loop. The suspension was collected, filtered through a sterile 40 μm pore size filter (BD Falcon, San Diego, CA, USA), and then counted and maintained in spore buffer at +4 °C for <1 week or skim milk at −20 °C for long-term storage. For counting and infection experiments, the suspension was centrifuged at 18.360 RCF at 4 °C for 5 min and the pellet was re-suspended in RPMI 1640 media. To exclude an impact of Dex on fungal growth, fungi were cultured in various Dex concentrations in the absence of cells. These analyses revealed no influence of Dex on germination and hyphenation as depicted in Figure S1.

### 2.7. Cytokine ELISA

At the end of the treatment and stimulation period (day 7), macrophages were stimulated with *A. fumigatus* at an MOI of 1 for 24 h. Culture supernatants were harvested and analyzed for cytokines including IL-1ß, TNFα, IL-10, IL-8, IL-15, and IL-6, using commercially available sandwich enzyme-linked immunosorbent assay kit (ELISA MAX^TM^ Deluxe Set, Biolegend, San Diego, CA, USA) specific for each cytokine as per manufacturer’s instructions. Absorbance was determined using the Biorad 680 microplate reader at 450 nm/570 nm filters (Biorad, Hercules, CA, USA).

### 2.8. HCS Analyses for Morphological Changes, Adherence Quantification and Mitochondrial Function

Glass-bottomed 96-well plates (Cell Carrier Ultra, Perkin Elmer, Waltham, MA, USA) were coated using 0.1% (*w*/*v*) Poly-l-lysin (Sigma-Aldrich, St. Louis, MO, USA) at a dilution of 1:10 in deionized water for 15 min to facilitate attachment and washed using Dulbecco’s Phosphate Buffered Saline (DPBS) (Sigma Aldrich, St. Louis, MO, USA). The wells were then seeded with macrophages (2 × 10^5^/200 µL) in respective media and left to adhere inside a humidified incubator at 37 °C/5% CO_2_ overnight. Following media removal, cells were stained in uninfected conditions, washed 3 times using DPBS, and fixed with 4% paraformaldehyde. HCS was done using the Operetta CLS^TM^ for visualization of stained cells and qualitative and quantitative image analysis using Harmony^TM^ software ( Perkin Elmer, Waltham, MA, USA). For mitochondria analysis, active mitochondria in live macrophages were stained with Mitotracker^®^ Orange CM-H2TMRos (1 μM) for 30 min at 37 °C/5% CO_2_. Nuclei were stained with Höchst or in some experiments Draq5 (both 1/1000). Fluorescent images were analyzed for intensity and data recorded as mean fluorescence intensity (i.e., total intensity/number of examined cells) for each macrophage per field. Morphological differences and adherence capacity were observed using inverted phase contrast and confirmed using confocal microscopy. Macrophages were stained using WGA (5 μg/mL) for 30 min at 37 °C. Nuclei were stained with Höchst 33.342 (1/1000). Adherence analysis was done using the Perkin Elmer-Harmony software containing a ready-made solution for cell quantification in high throughput.

### 2.9. Functional Assessment of Macrophage Ability to Prevent Phagocytosis, Conidia Germination, and Hyphae Growth Following Dexamethasone Treatment

HCS was used to assess immune cell dynamics in terms of antifungal activities of isolated macrophages against *A. fumigatus*. Therefore, macrophages were challenged using ds Red *A. fumigatus* at an MOI of 1 for 3, 6, 12, and 24 h at 37 °C/5% CO_2_. Cultures of conidia without any macrophages served as controls. Later, supernatants were removed, and the cells were washed extensively with ice-cold DPBS followed by staining and fixation with 4% PFA. Imaging plates were then stored in the dark until they were viewed in the microscope. Three-dimensional images as well as xyz stacks were acquired using Operetta CLS (PerkinElmer, Waltham, MA, USA) and analyzed using Harmony Software. Concurrently, 5 × 10^5 polarized treated and untreated macrophages were seeded onto FACS tubes for analysis of phagocytosis using flow cytometry. They were then challenged using ds Red *A. fumigatus* conidia at an MOI of 1 for 3 h. During these experiments, swollen conidia were obtained by incubating resting conidia in respective media at 37 °C for 3 h before infecting immune cells in order to enhance internalization. For ds Red *A. fumigatus* germination analysis at 12 h, cells were stained using WGA and Hoechst. For hyphae formation measurements at 24 h, calcofluor white rapid staining of chitin and cellulose on fungal cell membrane was done for imaging at an excitation of 340 nm and emission of 380 nm. Macrophage nuclei were counter-stained using Draq 5 far-red fluorescent DNA dye. Staining was carried out for 30 min at 37 °C followed by imaging without washing to avoid losing Dex-MDMs.

### 2.10. Intracellular Marker for Differentiation and PKM2 Enzyme Activity Assessment

To confirm the expression of macrophage lineage marker CD68, viable cells were first fixed using 4% PFA, permeabilized for 15 min using Perm/Wash buffer (BD Biosciences, San Diego, CA, USA) and stained for 30 min/4 °C using specific fluorophore-conjugated anti-human monoclonal Abs. They were then washed and fixed in 4% PFA. For PKM2 enzyme analysis, the cells were stimulated with *A. fumigatus* (24 h) at the end of treatment and stimulation period (day 7) and stained for PKM2 (Cell Signaling Technologies, Danvers, MA, USA) as described above.

### 2.11. Repolarization Assessment

Monocytes were stimulated for 7 days with 50 ng/mL rhGMCSF without Dexamethasone. Dexamethasone was then applied after 7 days at 0.1 µg/mL, 1 µg/mL, and 500 µg/mL for 24 h and 120 h. Stimulation of monocytes with 50 ng/mL rhGMCSF and 50 ng/mL rhMCSF served as M1 and M2 control, respectively.

### 2.12. Statistical Analysis

GraphPad Prism 8.0 Software (GraphPad Software Inc., San Diego, CA, USA) was used for statistical analysis. Unless stated otherwise, the one-way Analysis of Variance (ANOVA) was used to assess differences between treated and untreated groups and the student’s *t*-test was applied to analyze differences between treatment conditions against the control. All experiments were performed at least 3 times. For ELISA, data were expressed as mean of 2 duplicates and compared using students *t*-test (2-tailed paired). A *p*-value < 0.05 was considered significant for all statistical analysis.

## 3. Results

### 3.1. Dexamethasone Treatment Results in Significant Reduction in Macrophage Adherence Capacity

**Figure 1:**
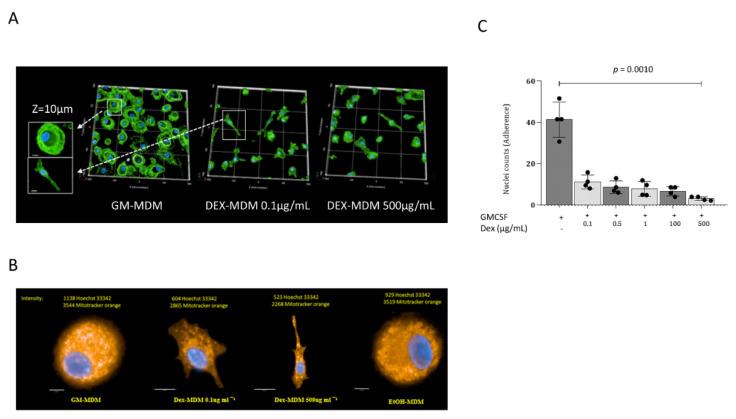
Dexamethasone changes morphology and adherence capacity of macrophages. Representative images show striking differences between untreated GM-MDMs (round morphology, higher adherence) vs. their Dex-treated counterparts (spindle-like morphology, less adherent). While Dex-MDMs illustrate the spindle-like morphology, in EtOH-treated control MDMs, there was no effect on morphology. (**A**) GM-MDMs (1 × 10^6^/mL) were treated with GM-CSF alone or in presence of GM-CSF and increasing concentrations of Dex (0.1 to 500 µg/mL) for 7 days. Thereafter, cells were fixed and stained using Wheat Germ Agglutinin (WGA) AF488 for cell surface and cytoplasm (green) and Höchst for nuclei (blue). Scale bar, 50 µm. (**B**) Confluence was quantified in an automated fashion before and after Dex treatment using the Operetta CLS Harmony Software (Perkin Elmer). The values were expressed as mean ± SD, demonstrating the change in confluence following treatment. The x-axis represents treatment conditions, and significance was calculated by ANOVA. (**C**) Mitochondria (orange) were stained using Mitotracker^®^ CM-H2TMRos and nuclei (blue) were stained using Höchst. Scale bar, 10 μm. All stainings were repeated at least three times, and a representative is depicted in the Figures.

In order to see if Dex treatment can impact macrophage survival when added during the monocyte/macrophage differentiation period, we first studied cell viability at physiologically relevant concentrations of the corticosteroid. At concentrations of ≤0.5 mg/mL, the cells remained viable throughout the differentiation period of 7 days when compared to control GM-CSF-differentiated MDMs. Significant cytotoxicity (57.94%) was observed at 1 mg/mL (*p* = 0.0218). However, more detailed analyses revealed that the observed cytotoxic effects were due to the solvent (EtOH) when applied at the corresponding levels of 4.8% (*p* < 0.0001) (Figure S2A–C). Despite the lack of cytotoxic effects attributable to Dex at the concentrations used, we observed significant alterations in the adherence capacity of macrophages differentiated in this manner as well as morphologic changes. While MDMs from healthy blood donors, differentiated in the presence of GM-CSF (GM-MDMs) for 7 days, exhibited a typical round phenotype and high confluence, Dex-/GM-CSF-differentiated MDMs (hereafter referred to as Dex-MDMs) lost their capacity for adherence and demonstrated a more spindle-shaped morphology at all tested Dex concentrations (0.1–500 µg/mL) (Figure 1A,B). The highest impact on adherence was observed upon differentiating the monocytes in the presence of 500 µL/mL Dex (*p* = 0.0010) (Figure 1C). Confluence was reduced by >80% when compared to the untreated control.

These results reveal that Dex exerts an impact on monocyte-to-macrophage differentiation with respect to their morphology and adherence capacity but not to their viability.

### 3.2. Dex-MDMs Significantly Change Their Profile of Surface Expression Markers

**Figure 2:**
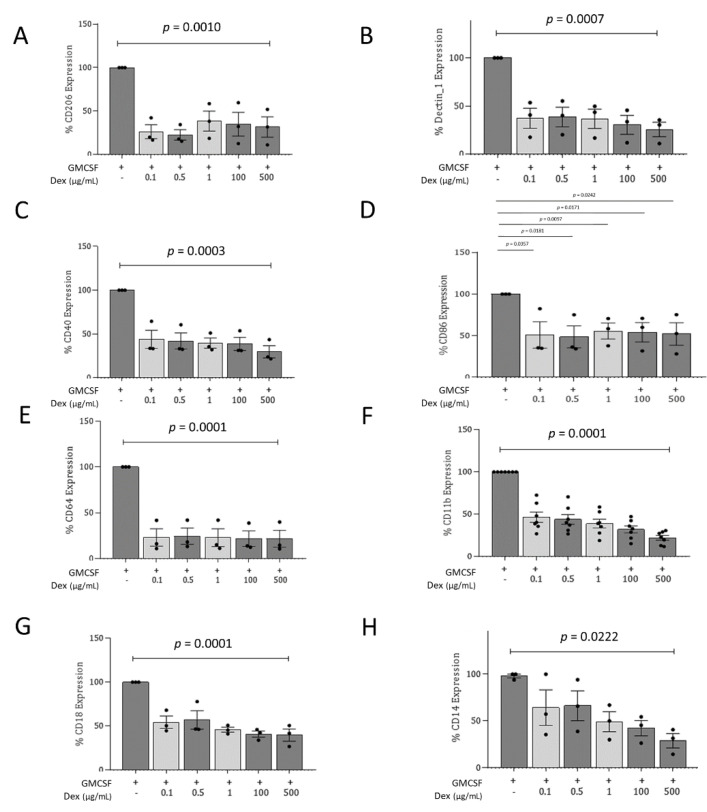
Dexamethasone impairs expression of pattern recognition receptors (PRRs) and co-stimulatory molecules, FcγRI, CR3, and CD14 on macrophages. Again, macrophages (1 × 10^6^/mL) were cultured for 7 days in the presence of GM-CSF alone or in combination with Dex (0.1 to 500 µg/mL). Flow cytometric analysis revealed that treatment significantly reduced the expression of (**A**) CD206, (**B**) Dectin-1, (**C**) CD40, (**D**) CD86, (**E**) CD64, (**F**) CD11b, (**G**) CD18, and (**H**) CD14. Graphs represent percentage expression of characteristic receptors expressed on macrophages. All experiments were independently repeated at least 3 times. Graphs representing mean fluorescence intensity (MFI) ± SD as well as a representative histogram plot of GM-MDMs, Dex-MDMs, or EtOH controls are illustrated in Suppl. Figure S3. *p*-values were calculated by one-way ANOVA.

To characterize Dex-MDMs in more detail, a comparison was made between Dex-MDMs and MDMs from healthy donors, differentiated in the presence of GM-CSF (GM-MDMs) or M-CSF (M-MDMs) for 7 days. These experiments revealed a significant decrease in various pattern recognition receptors (PRRs) such as CD206 (macrophage mannose receptor, MMR) and Dectin-1, the FcγR1 CD64, and co-stimulatory molecules CD40 and CD86 on Dex-MDMs, irrespective of the concentration applied during differentiation (Figure 2A–E, Figure S3A–E). A significant reduction in the expression of both molecules associated with the αMβ2 integrin complex (CD11b/CD18, complement receptor 3/CR3) was also observed following Dex treatment. These changes were highly significant (*p* < 0.0001) and concentration-dependent, being observable at day 7 of differentiation, in particular regarding CD11b expression (Figure 2F,G; Figure S3F,G). Downmodulation of CR3 correlated nicely with the reduced adherence capacity of Dex-MDMs (Figure 1). In addition, a significant impact on the expression levels of the CD14 surface marker was observed. This marker has been recently reported to promote a shift from Th1 to Th2 responses in the pulmonary system [37] (Figure 2H).

Therefore, the presence of Dex skews macrophages into M2-like phenotype properties.

### 3.3. Dex-MDMs Significantly Upregulate CD163

**Figure 3:**
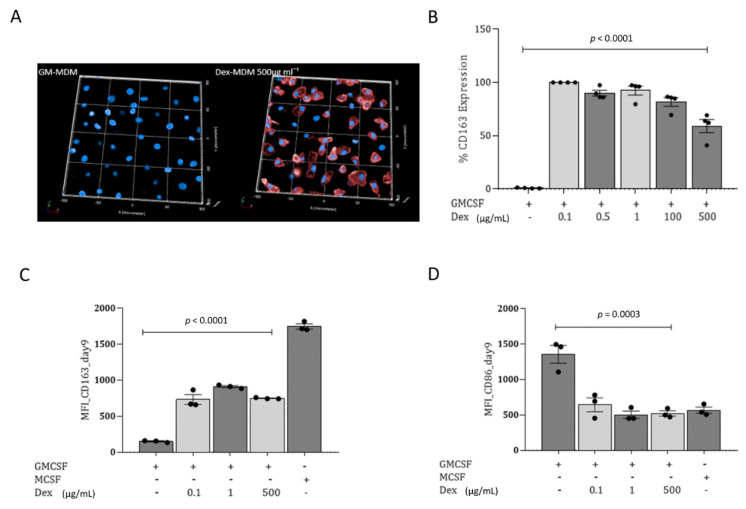
Dexamethasone upregulates the expression of the CD163 scavenger receptor. (**A**) α–human CD163 mAb-APC (red) and Höchst (blue) were applied for visualization purposes. HCS was performed and 3D image analyses done on GM- and Dex-MDMs. (**B**) Graph depicting upregulated expression of CD163 following 7-day treatment of GM-MDMs. Bars represent mean fluorescence intensity ± SD of n = 4 donors. The x-axis represents treatment conditions. GM-MDMs were treated with Dex for 24h post-differentiation period (7 days) and were repolarized into anti-inflammatory phenotypes depicted by upregulated expression of CD163 (**C**) and decreased expression of CD86 (**D**). Graphs demonstrate repolarization in three independent donors. Values are expressed as MFI ± SD, *p*-values indicated were calculated by one-way ANOVA.

In the next step, we evaluated the expression of CD163 (hemoglobin/haptoglobin receptor), a marker for alternatively activated M2 macrophages defined by anti-inflammatory and regulatory properties. CD163 was significantly higher in Dex-MDMs (Figure 3A,C) as well as in M-MDM (Figure 3B) compared to GM-MDMs (Figure 3A–C) as assessed by high content screening (HCS) and 3D image analyses (Figure 3A) or flow cytometry (Figure 3B,C). This upregulation on Dex-MDMs was even observed when macrophage differentiation was performed using GM-CSF only for 7 days and then adding the corticosteroid for another 24 h period (Figure 3C). This CD163 upregulation and repolarization from M1 to M2 macrophages by 24 h Dex treatment was accompanied by a concomitant downmodulation of the co-stimulatory marker CD86 (Figure 3D). Interestingly, there was no impact in the expression of the lineage marker CD68 (Figure S4), indicating that Dex did not prevent the differentiation of monocytes into macrophages.

Thus, Dex mediates repolarization of classically activated M1 macrophages, defined by antimicrobial and pro-inflammatory properties, into alternatively activated M2 macrophages, even when the corticosteroid is added short-term following an extended differentiation phase using GM-CSF.

### 3.4. During Monocyte-to-Macrophage Differentiation, Dexamethasone Significantly Changes Macrophage Metabolic Activity and Dampens the Pro-Inflammatory Immune Response

**Figure 4:**
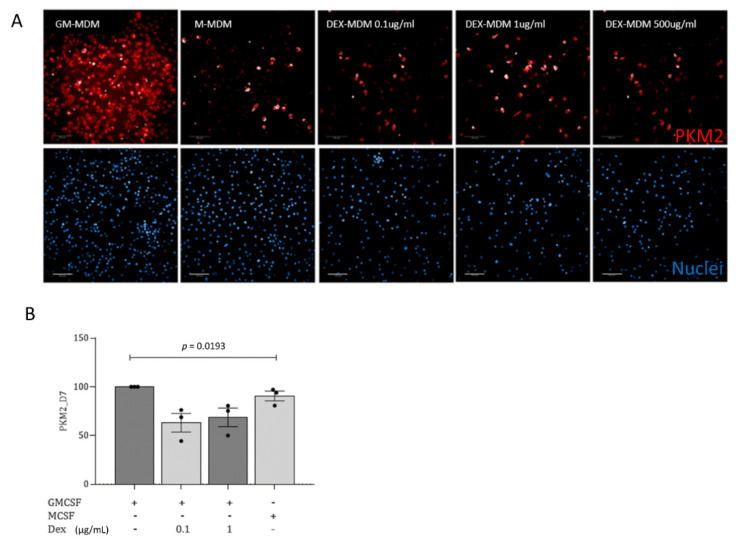
Dexamethasone decreases PKM2 activity in GM-MDMs. (**A**) PKM2 was visualized using HCS (Operetta CLS, Perkin Elmer) and the 20× water objective, NA 1.0. The images depict a downregulated PKM2 expression following treatment of GM-MDMs with 100 ng/mL, 1 µg/mL, and 0.5 mg/mL Dex. Scale bar, 100 μm. (**B**) Flow cytometric analyses in three independent donor cells also revealed that the presence of Dex during the monocyte-to-macrophage differentiation process significantly decreases PKM2 levels. M2-MDMs served as controls. Experiments were repeated independently three times, and statistical significance was evaluated using one-way ANOVA.

To see if the metabolic activity of macrophages is altered upon Dex treatment during differentiation, we next studied the expression levels of the enzyme Pyruvate kinase isoform M2 (PKM2) in GM-, Dex- and M-MDMs, which plays a rate-limiting role in metabolic activities in terms of facilitating glycolysis. We found a significant reduction in PKM2 activity when Dex was added during monocyte differentiation together with GM-CSF, compared to GM-MDMs (Figure 4A,B). The reduction mediated via Dex during differentiation was even enhanced in comparison to M-CSF-differentiated macrophages (Figure 4B).

We here demonstrate that Dex mediates a metabolic switch in macrophages, causing them to shift to an anti-inflammatory profile.

### 3.5. Dexamethasone Treatment of Macrophages Significantly Exacerbates Fungal Infection and Enhances Hyphenation during Aspergillus (A.) fumigatus Infection

**Figure 5:**
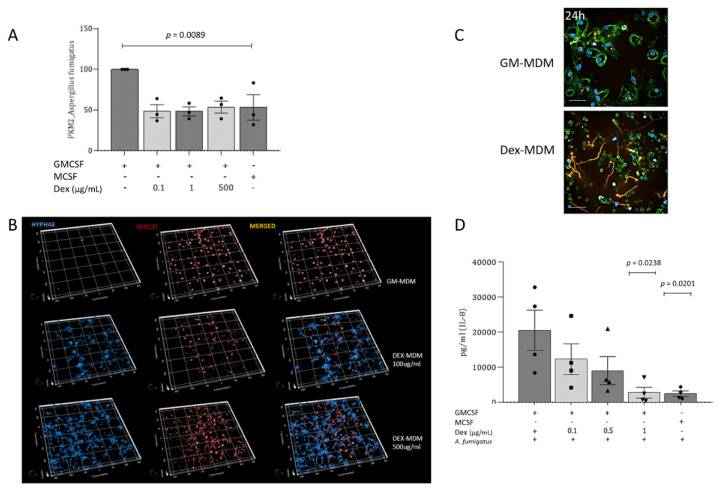
Fungal infection does not restore PKM2 levels in Dex-MDMs, and Dexamethasone treatment accelerates fungal growth by suppressing pro-inflammatory immune responses. (**A**) Upon treatment of the various MDM subsets (GM-, Dex, M-MDM) using *A. fumigatus*, PKM2 levels remained significantly lower in M- and Dex-MDMs, independently of the Dex-concentration used. M2-MDMs served as controls. Experiments were repeated independently three times, and statistical significance was evaluated using one-way ANOVA. (**B**) *A. fumigatus* was successfully suppressed in MDMs cultured in the presence of GM-CSF (M1), while Dex-MDMs were more prone to fungal infection and outgrowth. Nuclei were stained using Draq5 (red) and fungi using calcofluor (blue). The staining was independently performed 3 times. (**C**) Hyphal growth was only detectable in Dex-MDMs after overnight incubation, while GM-MDMs phagocytosed the fungal conidia (orange) but did not allow outgrowth. Nuclei were stained using Höchst (blue), cell surface using WGA (green). The experiment was repeated three times. Scale bar, 50 μm. (**D**) Impairment in IL-8 secretion was analyzed after stimulation with *A. fumigatus* in both Dex- and M-MDMs compared to IL-8 levels in *A. fumigatus*-exposed GM-MDMs. The most significant decrease in IL-8 production occurred in a Dex-concentration-dependent manner, and at the highest Dex-concentration tested (1 µg/mL), Dex-MDMs secreted as little IL-8 as M-MDMs upon fungal infection. The experiment was repeated four times due to donor-specific differences in cytokine secretion. All four experiments are depicted; statistical significance was calculated using ANOVA.

After characterizing in detail the effects of Dex on macrophage phenotype and functions, GM-, Dex-, and M-MDMs were infected with *A. fumigatus* conidia. Infection of GM- and Dex-MDMs with wild-type *A. fumigatus* conidia (MOI = 1) resulted in differential activation of PKM2, while PKM2 was strongly triggered in GM-MDMs; activation of PKM2 in Dex-MDMs was comparable to that in M-MDMs and significantly lower than *Aspergillus*-exposed GM-MDMs (Figure 5A). This further resulted in a significantly enhanced fungal burden in macrophages differentiated in the presence of the corticosteroid and GM-CSF as analyzed using calcofluor (Figure 5B). Three-dimensional image analyses revealed that calcofluor staining was only observable in macrophage cultures differentiated using Dex (100 and 500 µg/mL, respectively), whereas in GM-MDMs, it was not detectable and only marginal staining was apparent in vehicle-differentiated MDMs (EtOH-MDMs) (not shown). *A. fumigatus* germlings were already observable in Dex-MDMs after 3 h and 12 h (Figure S5) and following overnight incubation fungal hyphae were only detectable in Dex-, but not in GM-MDM (Figure 5C). In addition to the increased fungal growth, there was also a notably reduced secretion of the pro-inflammatory and antimicrobial cytokine IL-8 by Dex-MDMs (Figure 5D).

In summary, our data illustrate that corticosteroid treatment during monocyte-to-macrophage differentiation repolarizes GM-MDMs into anti-inflammatory M2-like MDMs not able to prevent fungal germination and hyphae formation.

## 4. Discussion

Glucocorticosteroids like Dexamethasone or Prednisolone have been suggested to play a role in aggravating fungal infection [16]. Therefore, we investigated the effect of immunosuppressive treatment on macrophage differentiation and function under the influence of the commonly prescribed steroid, Dexamethasone and its impact on fungal infection of macrophages. With mortality rates close to 90% in high-risk populations, there is an urgent need to understand at a mechanistic level why Dex promotes invasive aspergillosis, i.e., patients receiving long-term corticosteroid therapy [12]. To this end we characterized in detail the impact of Dex on macrophage phenotype and function and its role in promoting fungal infection. Following empirical determination of relevant Dex concentrations to be applied, we observed that under the influence of Dex, macrophage survival and the ability to differentiate from classical CD14+ monocytes remained unimpaired (non-classical CD16+ CD14- [38] were excluded from the study). These differentiation and cell survival outcomes were corroborated by the unaffected expression of the CD68 lineage-associated surface receptor and were in accordance with previously reported results published by Kuppermann and colleagues who analyzed Dexamethasone’s effect on retinal cells [25].

Despite the presence of GM-CSF during monocyte-macrophage differentiation, Dex treatment significantly upregulated the M2-associated surface marker, CD163, at all concentrations tested. This Dex-induced CD163 upregulation points towards macrophage polarization and repolarization from a pro-inflammatory M1 to an anti-inflammatory or pro-resolving M2 phenotype. CD163 is a 130-kDa membrane protein exclusively expressed by monocytes and macrophages and commonly associated with anti-inflammatory conditions [39,40,41]. Simultaneously with CD163 induction, we observed consistent morphological changes characterized by a more elongated phenotype, which is also commonly associated with the M2 Macrophage variant [7]. Morphological changes due to long-term Dexamethasone treatment have been previously reported in porcine hepatocytes [19] and the human liver cancer cell line HepG2 [42], which indicates that this effect is not exclusive to immune cells. The treated cells also exhibited a reduced ability to attach to extracellular matrices accompanied by a downregulation in PRR expression. Since GM-CSF is a well-known immune system modulator administered in conjunction with other cytokines such as IFN-γ, antifungals, or monoclonal antibodies to enhance anti-fungal activities of innate phagocytic cells [43], we used GM-CSF as the pro-inflammatory promoter for this study. When applying GM-CSF in combination with Dex, the impact of the corticosteroid outweighed the pro-inflammatory features of GM-CSF, resulting in opposing effects being observed on several markers, including chitin and β-glucan-associated pattern-recognition receptors, MR (CD206) and Dectin–1 (CLEC-7A), when compared to cells treated with GM-CSF alone. Since these germline-encoded structures are crucial for recognition of conserved PAMPs [44] and mediation of apoptotic cell endocytosis [45], they also ensure the mounting of inflammatory responses against invaders [46,47]. Therefore, an alteration in their expression profile is crucial to steady-state macrophage functions. CD206 has been shown to be elevated on the surface of murine M2 but not on human macrophages, as seen by us and others [6]. FcγR1 (CD64), an essential receptor recognizing IgG-complexed antigens [5,48] was also significantly downregulated by the steroid, which implies that the drug also affects antibody-mediated immune complex signals.

Dex treatment during monocyte-to-macrophage differentiation also resulted in the reduction of CD11b and CD18, adhesion molecules essential for vascular migration of leukocytes to inflammatory areas [43,49]. Our data showing a significantly reduced macrophage adherence capacity due to Dex treatment can therefore be interpreted as being a consequence of integrin molecule alterations resulting in reduced lamellipodia formation and ineffective functional activities when it comes to migration and engulfment of pathogens. Our results are consistent with previously published reports demonstrating that Dex-treated macrophages lacked adhesion signaling complexes referred to as podosomes [48]. Dex has also been reported to decrease the expression of adhesion-associated surface markers on the monocytes and neutrophils of neonates following in vivo treatment [50] as well as on human eosinophils [51]. These morphological and phenotypic alterations described herein point towards a repolarization of M1- into M2-like macrophages being induced by long-term Dex treatment, also in the presence of GM-CSF. Previously published studies implicate phenotypic differences as a result of changes in metabolic activities [52], but information on the impact of corticosteroids on macrophage metabolism is limited [53].

PKM2 exists as a tetramer that converts phosphoenolpyruvate (PEP) into pyruvate and a dimer that is a transcriptional coactivator for pro-inflammatory cytokines [54]. The dimeric form is known for its ability to attach to the mitochondrial membrane and maintain its function [55]. In active macrophages, this isozyme is critical for ATP synthesis through aerobic glycolysis [56], which results in metabolic remodeling during inflammation [57]. The pyruvate generated through the PKM2-associated glycolytic process enters mitochondria, where it is oxidatively decarboxylated by pyruvate dehydrogenase (PDH) to produce Acetyl-CoA, initiating the Krebs cycle [58]. Porcine hepatocytes undergoing long-term Dex treatment showed significant glycogen degeneration [19]. Since manipulation of mitochondrial functions has also been reported as one of the evasion mechanisms employed by various pathogens to ensure their survival [59], fungal pathogens might take advantage of Dex-mediated dysregulation of mitochondria to thrive in their growth, germination, and survival as shown herein. Recent studies showed that upregulation of the glycolytic pathway is a key mechanism for pro-inflammatory activities of macrophages against fungal pathogens [3,31]. In addition, lipopolysaccharide, a well-known stimulator of metabolic activities in macrophages, resulted in higher expression of the glycolysis-associated pyruvate kinase PKM2 [32] and induction of a pro-inflammatory signature [60]. PKM2 downregulation interfered with immune cell activation processes and limited inflammation [3], which might be counterproductive during invasive aspergillosis [12]. Here, we found that *A. fumigatus* takes advantage of the Dex-mediated macrophage switch, leading to extensive germination into full-fledged hyphae within 24 h. This is a feature that has been previously observed in clinical settings and mice experiments for which accumulation of mycelia is the principal characteristic indicating disease progression [16,61]. Hyphae overgrowth, observed in our in vitro study, nicely correlates with reports of increased tissue invasion by fungi. It has also been previously reported that in healthy individuals, 90% of conidia are killed after a day [62], a finding that we corroborated in GM-MDMs by demonstrating that they efficiently ingested fungal conidia and prevented hyphenation. Further reports of enhanced pathogen survival and replication in environments rich in M2 macrophages when compared to M1 macrophages have been made [7]. Therefore, the observed increase in hyphal growth could also be indicative of the changing microenvironment due to Dex-induced macrophage polarization. Taken together, repolarization of macrophages following corticosteroid treatment and concomitant switch to an anti-inflammatory phenotype might play a prominent role in triggering invasive Aspergillosis due to suppression of innate immunological responses necessary to combat extensive fungal outgrowth.

## Figures and Tables

**Table 1:** Antibodies used.

Antibody/Stain	Source	Cat Number	Concentration Applied
Alexa Fluor^®^ 488 anti-human CD11b	Biolegend, San Diego, CA, USA	301318	80 μg/mL
Alexa Fluor^®^ 488 anti-human CD40	Biolegend, San Diego, CA, USA	334318	40 μg/mL
Alexa Fluor^®^ 488 anti-human CD86	Biolegend, San Diego, CA, USA	305318	5 μL/test
Alexa Fluor^®^ 488 anti-human CD11b	Biolegend, San Diego, CA, USA	301318	5 μL/test
Alexa Fluor^®^ 488 anti-mouse CD11b	Biorad, Hercules, CA, USA	MCA74A488	10 μg/mL
Alexa Fluor^®^ 488 Wheat Germ Agglutinin	Biotium, Hayward, CA, USA	29022-1	5 μg/mL
Alexa Fluor^®^ 647 anti-human CD163	Biolegend, San Diego, CA, USA	326508	10 μg/mL
Alexa Fluor^®^ 647 anti-human CD369	BD Pharmingen, Franklin Lakes, NJ, USA	564855	5 μL/test
APC anti-human CD18	Biolegend, San Diego, CA, USA	302114	30 μg/mL
APC anti-human CD19	Biolegend, San Diego, CA, USA	302212	10 μg/mL
APC anti-human CD68	Biolegend, San Diego, CA, USA	333809	5 μL/mL
APC anti-human CD86	BD Pharmingen, Franklin Lakes, NJ, USA	555660	5 μL/test
BV421 anti-human CD86	Biolegend, San Diego, CA, USA	307636	5 μL/test
Draq 5	Biostatus, Loughborough, LEICS; UK	DR51000	5 nM
Calcofluor White Stain, Millipore	Sigma-Aldrich, St. Louis, MO, USA	18909	NEAT
FITC anti-human CD3	BD Pharmingen, Franklin Lakes, NJ, USA	555339	10 μg/mL
FITC anti-human CD64	BD Pharmingen, Franklin Lakes, NJ, USA	555527	20 μL/test
FITC anti-human CD206	BD Pharmingen, Franklin Lakes, NJ, USA	551135	20 μL/test
Ghost Dye^TM^ Viability	Tonbo Biosciences, San Diego, CA, USA	13-0870	10 μL/mL
Hoechst 33342	Sigma-Aldrich, St. Louis, MO, USA	B1155	2 μg/mL
Mitotracker^®^ Orange CM-H_2_TMRos	Invitrogen, Carlsbad, CA, USA	M7511	100–500 nM
PE anti-human CD14	Biolegend, San Diego, CA, USA	301806	40 μg/mL
PE anti-human CD86	Biolegend, San Diego, CA, USA	305406	20 μg/mL
PE anti-human CD163	Biolegend, San Diego, CA, USA	333606	40 μg/mL
PE anti-human CD206	BD Pharmingen, Franklin Lakes, NJ, USA	555954	2.5 μL/test
PE anti-human PKM2 (D78A4)	Cell Signaling Technology, Danvers, MA, USA	983675	2 μg/mL
Per CP Cy5.5 anti-human CD14	BD Bioscience, San Diego, CA, USA	562692	5 μL/test

## Supplementary Materials

The following are available online at https://www.mdpi.com/2309-608X/7/2/70/s1.
