# Supplementary material for: Dexamethasone Promotes Aspergillus fumigatus Growth in Macrophages by Triggering M2 Repolarization via Targeting PKM2
Source: J Fungi (Basel). 2021 Jan 20;7(2):70. doi: 10.3390/jof7020070 (PMC7909285; doi:10.3390/jof7020070)

Suppl. Figure 1 | In the absence of macrophages Dexamethasone did not affect germination of *Aspergillus fumigatus*

A. fumigatus was cultured in the presence of different concentrations of Dexamethasone for 12h. Dexamethasone did not prevent fungal outgrowth at any concentration applied. dsRed strain of *A. fumigatus* (seen in orange) germinated into germlings and hyphae despite treatment.

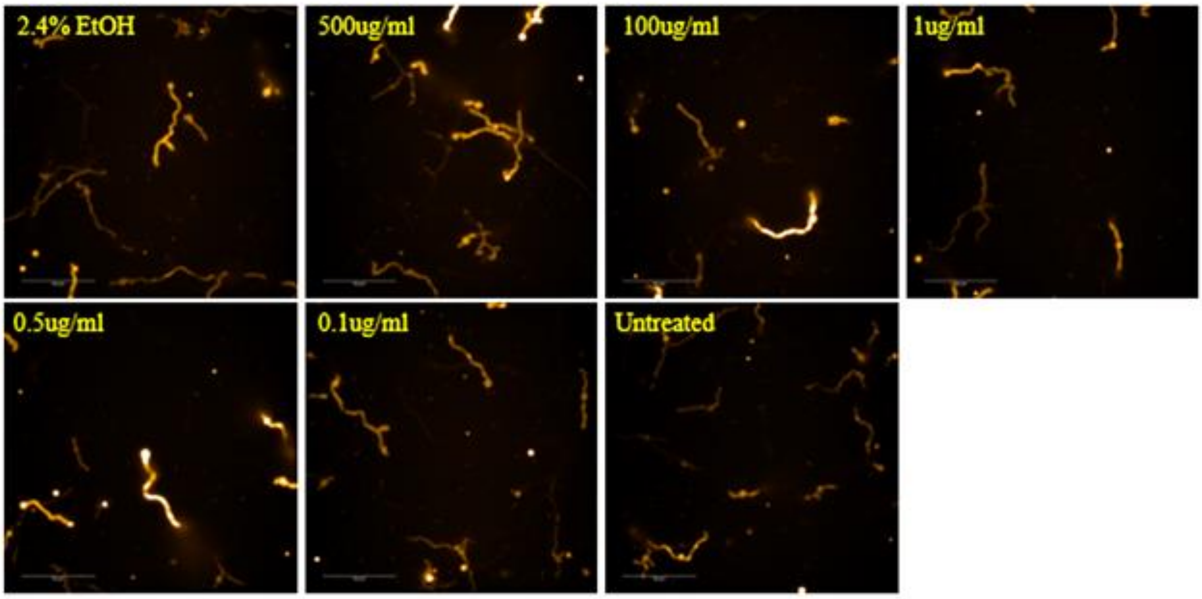

Suppl. Figure 2 | Macrophage viability is not affected by Dex treatment. Quantitative assessment of macrophage viability was done using flow cytometry and the results confirmed using the Trypan blue exclusion method at T_{168}. The graphs were expressed as mean ± SD demonstrating the change in cell survival following 7 - day treatment with Dex. DMSO treated cells were included as a dead cell control and 2.4% EtOH treatment was also applied to exclude unspecific effects. (A) Dex treatment of GM-MDMs (B) Dex treatment of THP1 cells (C) EtOH treatment of THP1 cells.

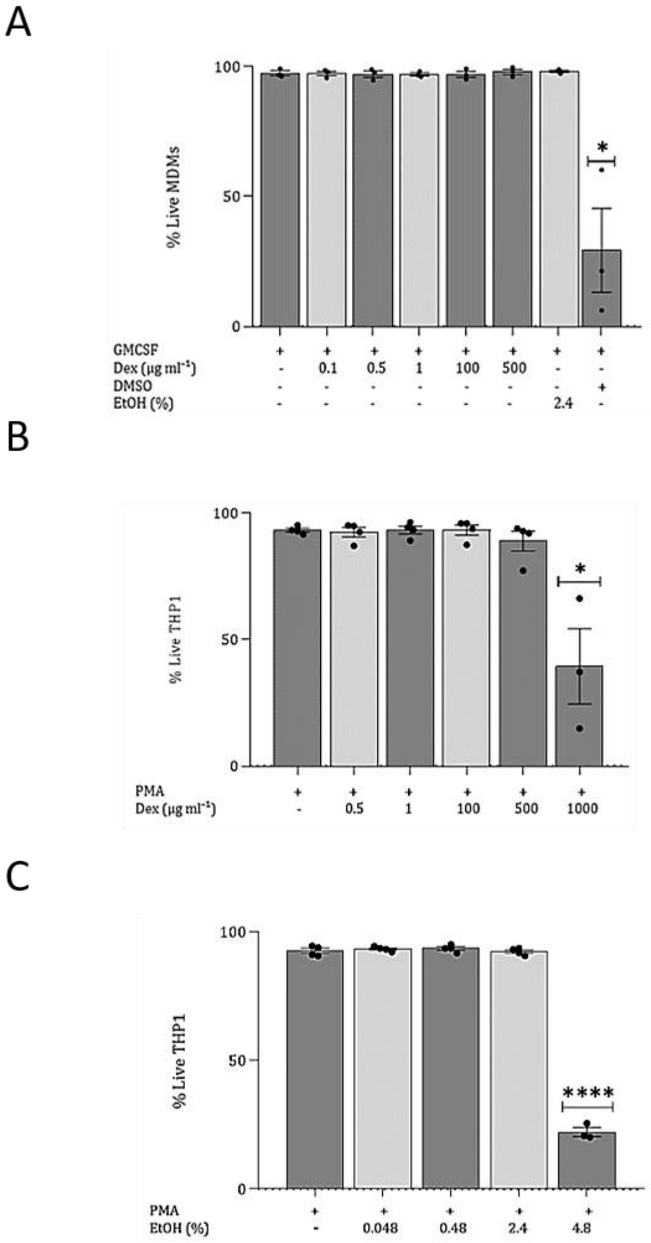

Suppl. Figure 3 | Dexamethasone impairs expression of PRRs, co-stimulatory molecules, FcγRI, CR3 and CD14 on macrophages. Macrophages (1 x 10^6/ml) were cultured for 7 days in the presence of GM-CSF alone or in combination with Dex (0.1 to 500 µg/ml). Flow cytometric analysis revealed that treatment significantly reduced the expression of (A) CD206, (B) Dectin-1, (C) CD40, (D) CD86, (E) CD64, (F) CD11b, (G) CD18, and (H) CD14.

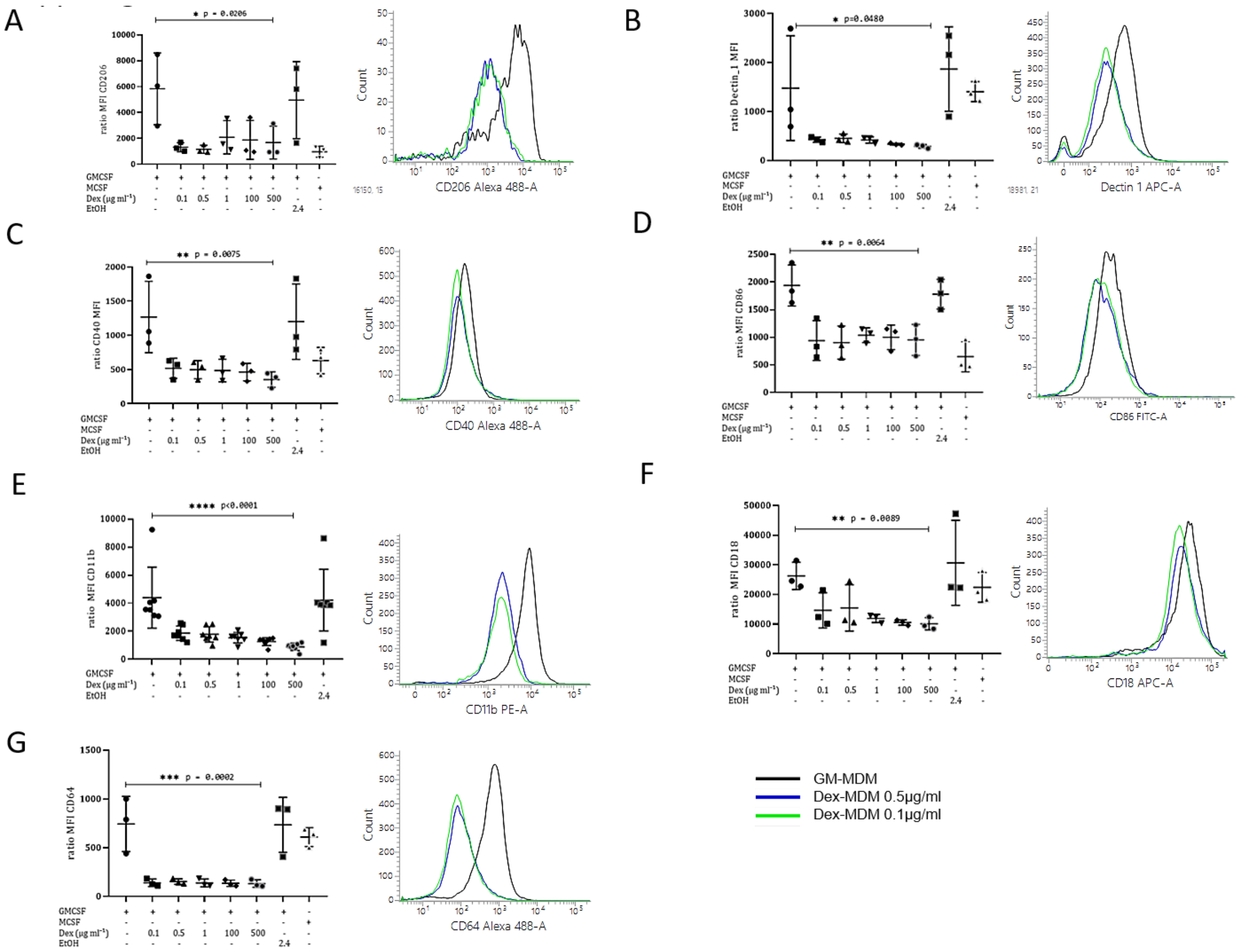

(Left) Graphs represent mean fluorescence intensity (MFI) ± SD as well as (right) one representative histogram plot of GM-MDMs, Dex-MDMs or EtOH (2.4%, corresponding to the highest EtOH concentration used at a Dex concentration of 500µg/ml) control macrophages. All experiments were independently repeated at least 3 times. Graphs representing in Suppl. Fig. 2. p-values were calculated by one-way ANOVA.

Suppl. Figure 4 | CD68 is not changed upon treatment with various Dex concentrations.

Macrophages (1 x 10^6/ml) were cultured for 7 days in the presence of GM-CSF alone or in combination with Dex (0.1 to 500 µg/ml). Flow cytometric analysis revealed that Dex treatment does not change CD68 expression levels independent on the concentration used. At least 3 independent experiments were performed, the results were statistically non-significant.

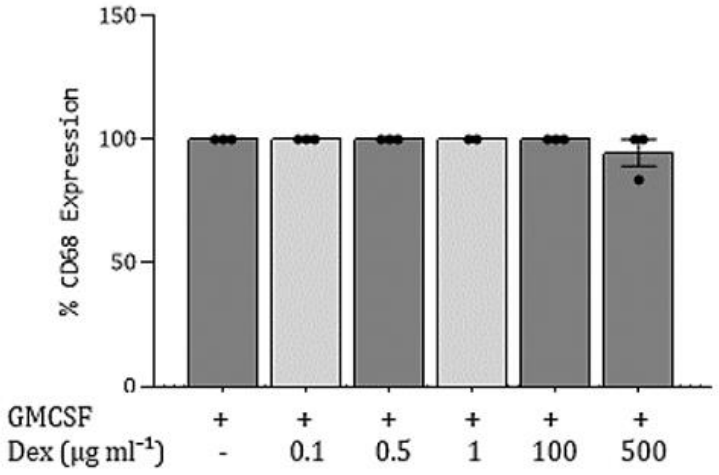

Suppl. Figure 5 | Dex-MDMs are more prone to fungal infections.

A. fumigatus was successfully suppressed in MDMs cultured in the presence of GM-CSF (M1) after 3h and 12h, while Dex-MDMs were more prone to fungal infection and outgrowth. Nuclei were stained using Höchst (blue), MDM surface using WGA (green) and a dsRed strain of *A. fumigatus* was used to monitor fungal growth (orange).

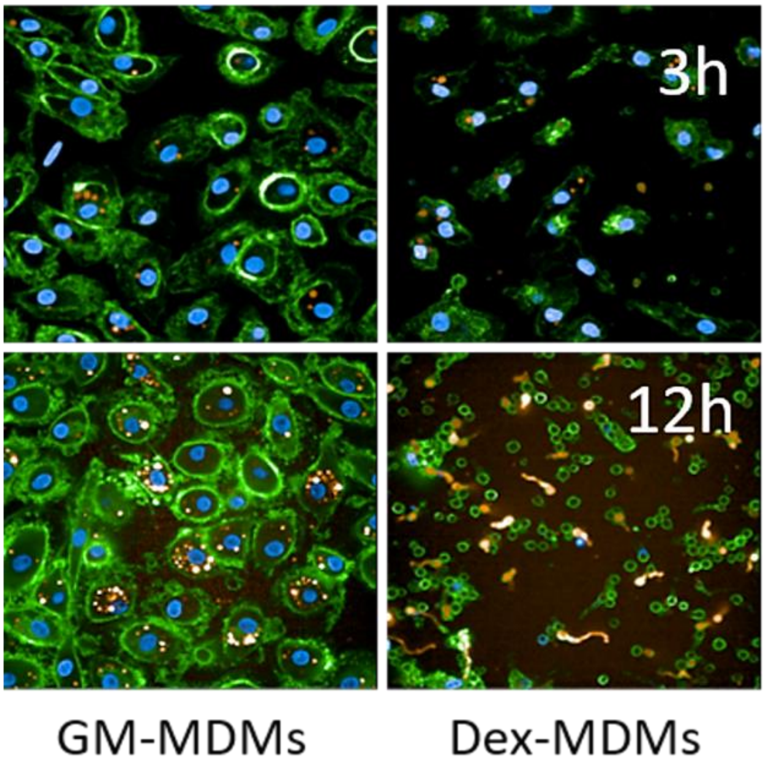

Supplement: Supplementary file 1 [file jof-07-00070-s001.pdf]
